# Correction: Frontal cortex tracks surprise separately for different sensory modalities but engages a common inhibitory control mechanism

**DOI:** 10.1371/journal.pcbi.1007420

**Published:** 2019-10-07

**Authors:** The PLOS Computational Biology Staff

There is an error within the equation on page 6 of the pdf (under the heading ‘Experimental design and statistical test (EEG analysis)).

Currently the equation reads:

$${Surprise}_{i}={log}_{2}\left(\frac{p_{unexpected\_cue}(1\ldots n)}{p_{unexpected\_cue}(1\ldots i-1)}\right)$$

It should read:

$${Surprise}_{i}={log}_{2}\left(\frac{p_{unexpected\_cue}(1\ldots i)}{p_{unexpected\_cue}(1\ldots i-1)}\right)$$
