# Supplementary figures and images for: Frontal cortex tracks surprise separately for different sensory modalities but engages a common inhibitory control mechanism
Source: PLoS Comput Biol. 2019 Jul 29;15(7):e1006927. doi: 10.1371/journal.pcbi.1006927 (PMC6687204; doi:10.1371/journal.pcbi.1006927)

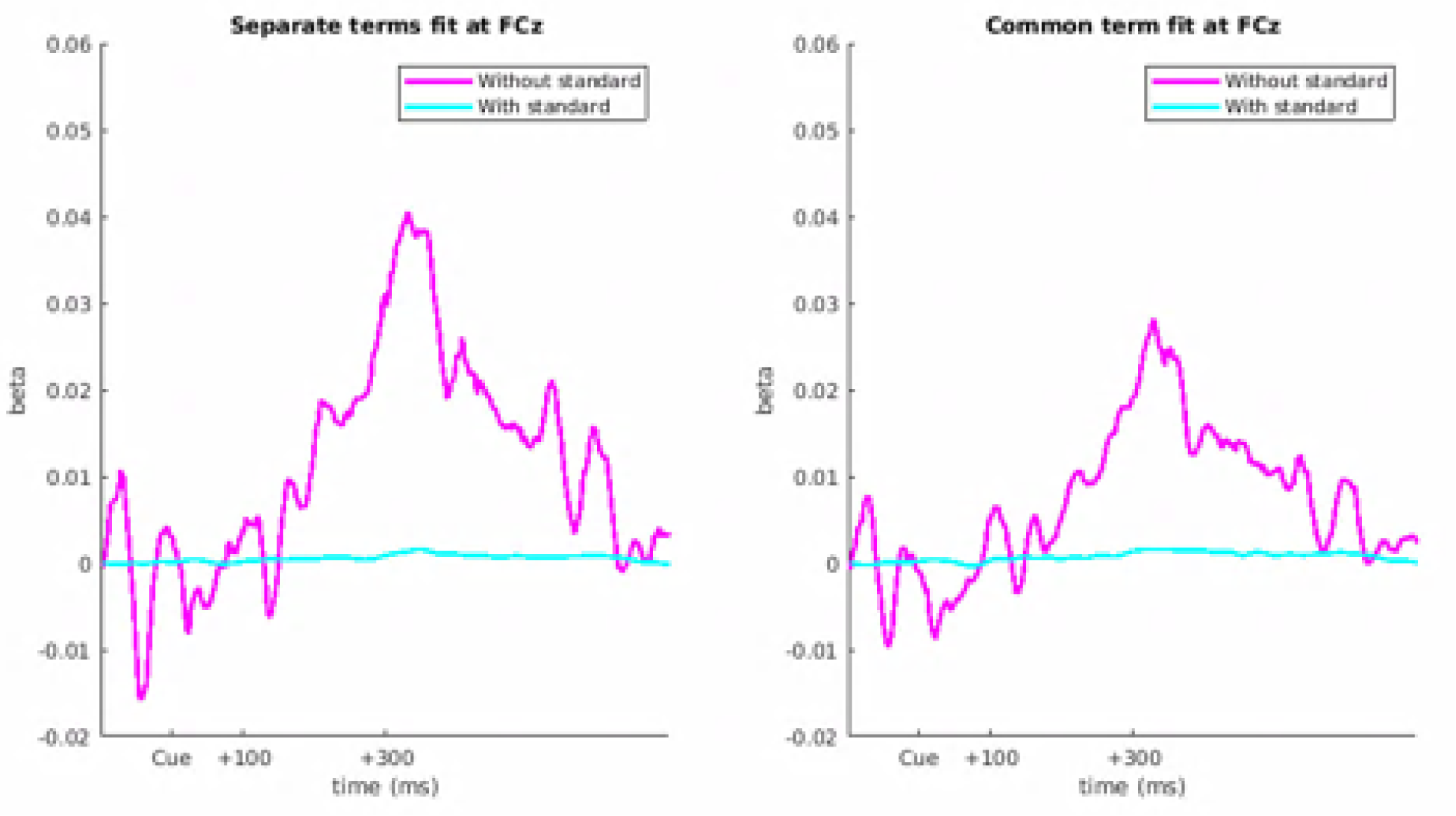

Supplement: S1 Fig — Fits are from electrode FCz, the centroid of positive significant model fit in the main analyses. As is evident, inclusion of the standard trials leads to a severe reduction in model fit for both models, almost certainly resulting from the inclusion of a large amount of near-zero values for the surprise term stemming from the standard-cue trials. (TIF) [file pcbi.1006927.s001.tif]

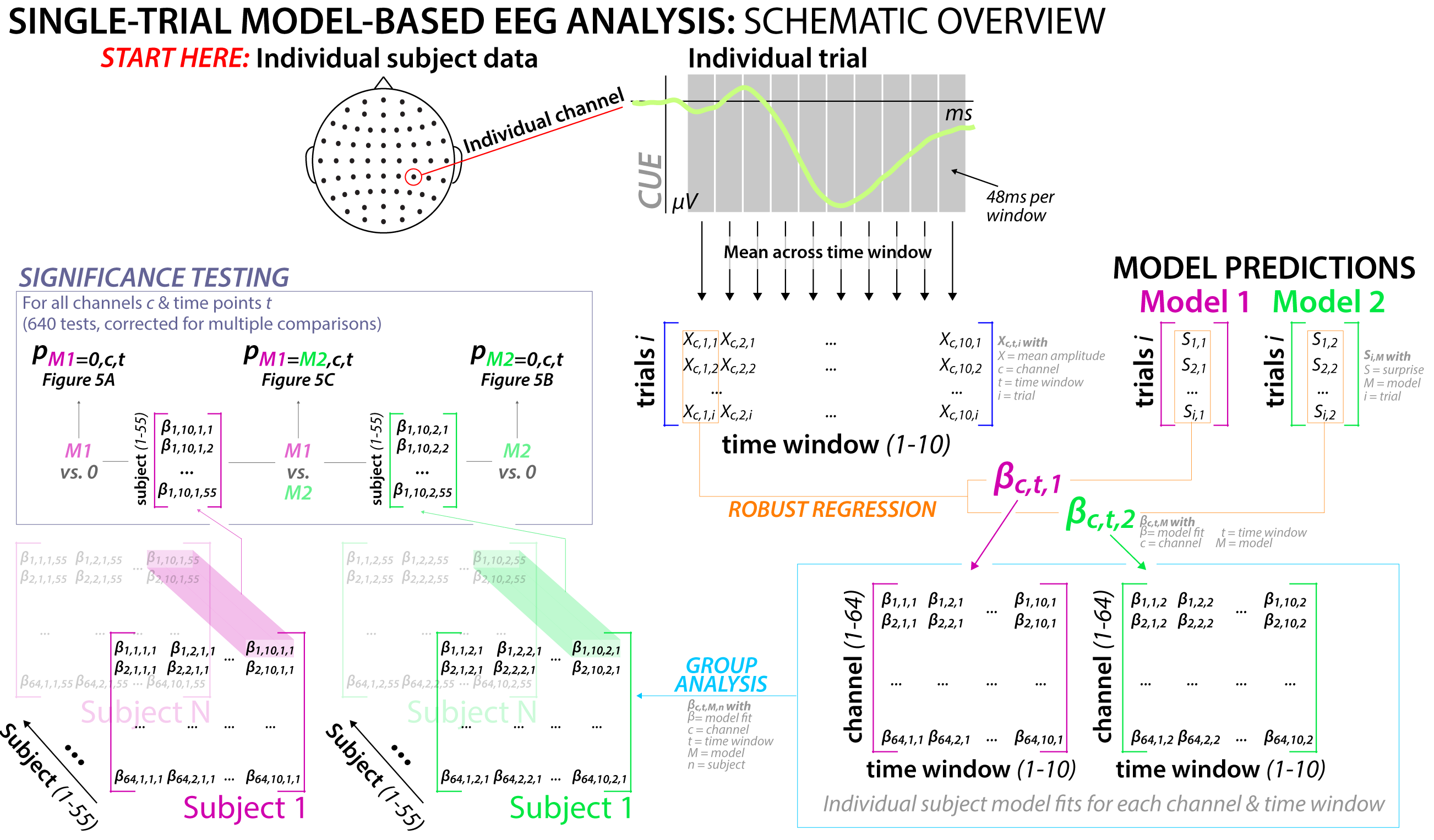

Supplement: S2 Fig — Clockwise from the top-left: For each individual channel, the trial-by-trial event-related response was averaged within 10 consecutive time windows following onset of an unexpected cue. This resulted in a matrix of 48 trials by 10 time windows of EEG amplitude values for each subject (one for each channel; blue brackets). Each subject’s individual model terms for both models (pink and green brackets on the top right) were then correlated with each of the trial-vectors for each time window using robust regression (orange line). The resulting beta values were stored in one channel by time window matrix for each subject and model (bottom right). These beta weights were subjected to group-level analyses across subjects (bottom left), with each channel by time window combination (640 unique combinations per model) tested against 0 for each model separately (purple box), with paired samples t-tests using subject as the random factor. (TIF) [file pcbi.1006927.s002.tif]

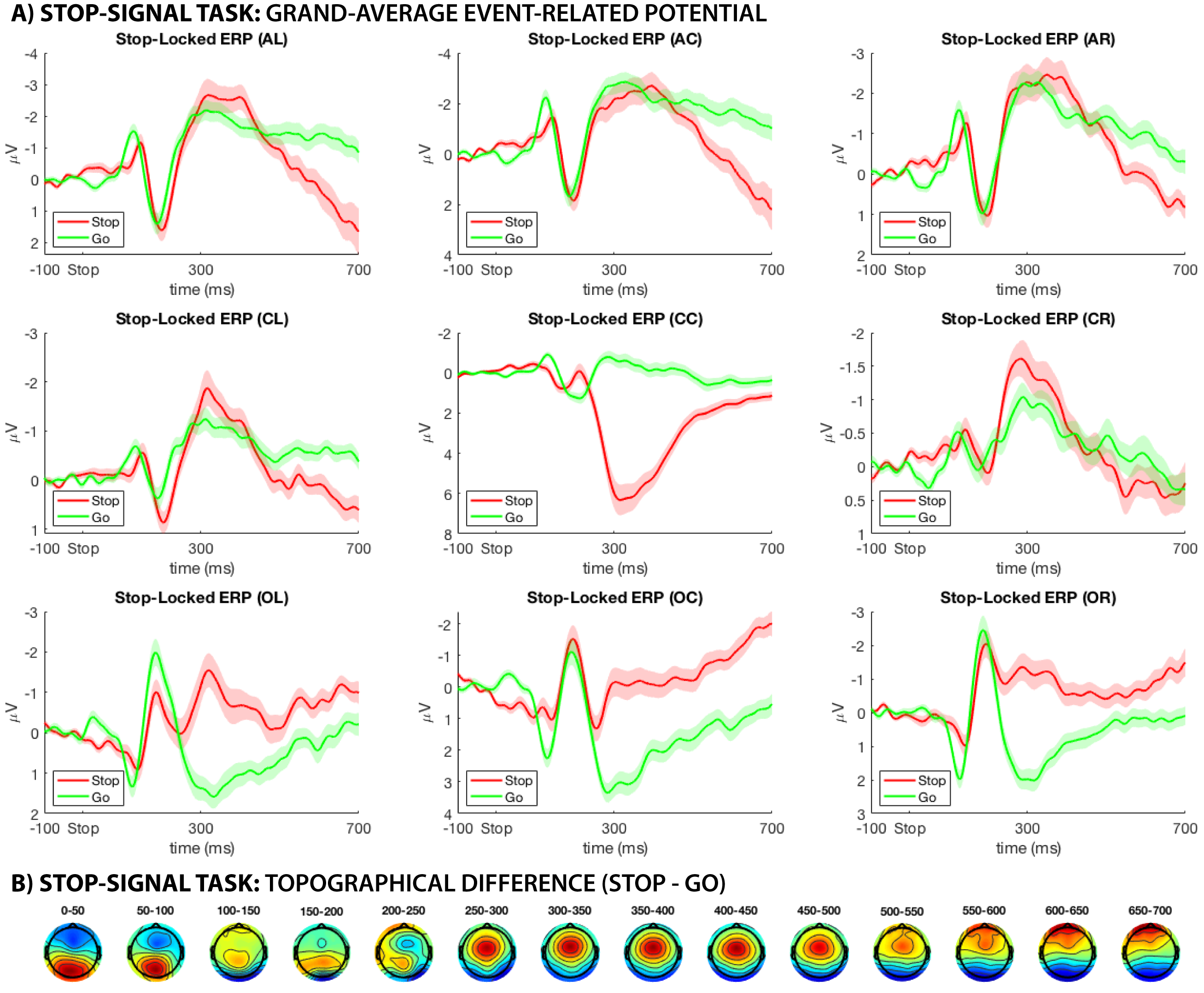

Supplement: S3 Fig — A) Time-courses at 9 differences scalp sites (AL = Anterior left electrode cluster, AC = Anterior central electrode cluster, AR = Anterior right electrode cluster; C* = central; O* = occipital). Shaded area represents the standard error of the mean ERP. B) Topographical representation during post-event time-periods (in ms). (TIF) [file pcbi.1006927.s003.tif]

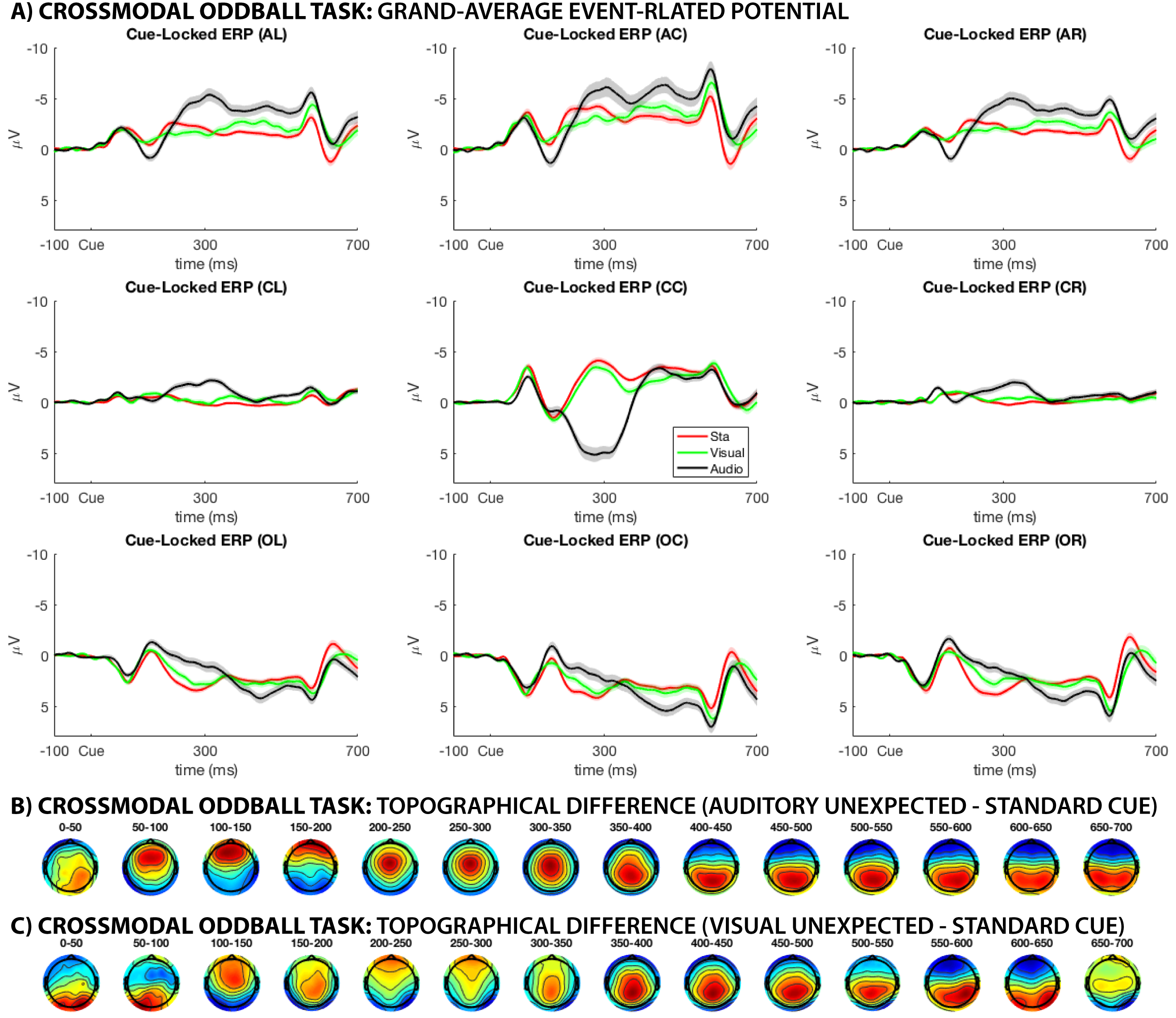

Supplement: S4 Fig — A) Time-courses at 9 differences scalp sites (AL = Anterior left electrode cluster, AC = Anterior central electrode cluster, AR = Anterior right electrode cluster; C* = central; O* = occipital). Shaded area represents the standard error of the mean ERP. B) Topographical representation during post-event time-periods (in ms) for the difference wave between unexpected auditory cues and standard cues. C) As B, but for unexpected visual cues. (TIF) [file pcbi.1006927.s004.tif]
